# Evaluation of prenatally diagnosed fetal sacrococcygeal teratomas: A case series of seventeen pregnancies from South-central Turkey

**DOI:** 10.4274/tjod.galenos.2020.68812

**Published:** 2020-10-02

**Authors:** Mehmet Özsürmeli, Selim Büyükkurt, Mete Sucu, Erol Arslan, Selahattin Mısırlıoğlu, Çiğdem Akçabay, Masum Kayapınar, Süleyman Cansun Demir, İsmail Cüneyt Evrüke

**Affiliations:** 1 University of Health Sciences Turkey, Kocaeli Derince Training and Research Hospital, Clinic of Obstetrics and Gynecology, Kocaeli, Turkey; 2 Çukurova University Faculty of Medicine, Department of Obstetrics and Gynecology, Perinatology Unit, Adana, Turkey

**Keywords:** Prenatal diagnosis, teratoma, sacrococcygeal region

## Abstract

**Objective:**

To evaluate sacrococcygeal teratoma (SCT) cases according to associated cardiac, extracardiac, and chromosomal anomalies in the prenatal period, and to review their outcomes.

**Materials and Methods:**

Data of pregnancies with a prenatal diagnosis of SCT between 2009 and 2019 were retrospectively reviewed.

**Results:**

One ongoing pregnancy was excluded. There were five medically terminated cases, three due to severe heart failure and the remaining two due to additional congenital defects. Two infants who had heart failure due to hyperdynamic flow died in the neonatal period. Nine infants are well and alive at the time of writing.

**Conclusion:**

When a lesion is detected in the sacrococcygeal region during fetal sonography, the differential diagnosis should be made with an appropriate evaluation with emphasis on a possible diagnosis of fetal SCT. Tumor growth and heart failure should be monitored with serial scans when SCT has been diagnosed prenatally.

**PRECIS:** In present study, we evaluated prenatally diagnosed cases with sacrococcygeal teratoma and associated anomalies.

## Introduction

Sacrococcygeal teratomas (SCT) are one of the most common congenital tumors with an incidence of 1/35,000 to 40,000 of live births^(1,2)^. The teratomas detected in the perinatal period mostly derive from pluripotent primitive stem cells in Hensen’s node in the sacrococcygeal region^(3,4)^. Teratomas are classified histologically as mature and immature, and immature elements are composed of primitive neuroglial tissues^(5)^.

**Figure 1:**
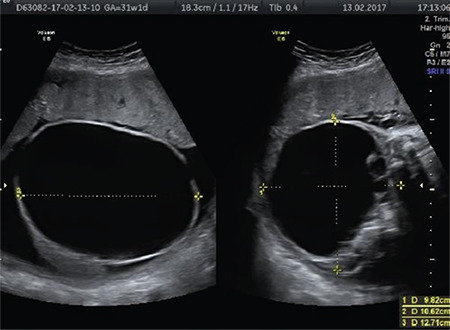
A case of sacrococcygeal teratoma with a cystic component originating from the sacrococcygeal region. The absence of intracranial findings excludes the diagnosis of spina bifida

Altman et al.^(6)^ described four types of SCTs according to anatomic location. Type 1 tumors are the most common with the majority of the tumor growing outward. In type 2 and type 3 tumors, the tumor has grown into the pelvis and out of the pelvis, respectively, with type 3 tumors growing more extensively into the pelvis. Type 4 tumors are entirely located in the pelvis. According to this classification, the best prognosis is in type 1 tumors^(6)^. Type 1, 2, and 3 tumors can be seen externally because they grow as an exophytic mass (Figure 1). Type 1, 2, and 3 tumors are easier to diagnose in both the prenatal and neonatal periods and have low malignant potential^(7)^. Type 4 tumors are usually diagnosed in the postnatal period and have higher potential for malignancy^(8)^.

SCTs appear as irregular thick-walled masses with cystic and solid components on ultrasound imaging^(3,9,10)^ and should be distinguished from spina bifida. Spina bifida has a significant bone defect and intracranial findings, which tends to be a higher level of the spine. Differential diagnosis includes myelocystocele, lipoma, hamartoma, hemangioma, lymphangioma, and ependymoma in fetal sacrococcygeal region masses^(11)^.

The prenatal course of SCTs is generally unpredictable. However, fetal heart failure due to fetal anemia and high blood flow into the mass can cause fetal hydrops, polyhydramnios, and preterm delivery^(12,13)^. SCTs have become much more detectable in the prenatal period with the increased use of ultrasound scan. Close monitoring and serial ultrasound imaging are supposed to identify some fetuses requiring fetal intervention and preterm birth.

The present case series from a single institution throughout a 10-year-period aimed to assess the characteristics and short- to medium-term follow-up of prenatally diagnosed cases of SCT.

## Materials and Methods

This retrospective study was conducted at Çukurova University Hospital (academic tertiary referral center) Prenatal Ultrasound Unit. All women diagnosed as having fetal SCT from January 2009 to September 2019 were analyzed. Data were collected from the digital patient archiving system. All pregnant women were informed, and written content was obtained. The study was subject to local ethics committee approval (approval no: 14, date: 5.10.2018).

All of the sonography evaluations were performed by one of the seven authors, using a convex volumetric probe (RAB 6-D 2-7 MHz and RAB2 5L). In general, fetal anatomy scans are performed between 18-22gestational weeks in the present clinic. The authors also evaluate potential fetal anomalies referred from other centers.

When a heterogeneous mass was seen in the sacrococcygeal region, a differential diagnosis was made. The diagnosis of spina bifida was excluded by demonstrating the continuity of the spinal canal. Color and power Doppler were used to determine blood flow into the mass. Serial ultrasonographic examinations were performed to detect hyperdynamic heart failure, development of fetal hydrops, and tumor growth. Routine fetal karyotyping was not recommended.

Neonatal outcomes were obtained from electronic medical reports, or the family was contacted by phone call. The antenatal findings of all cases with neonatal loss and termination of pregnancy were confirmed with autopsy examinations, except for first trimester terminations.

### Statistical Analysis

The study is the case series. Statistical analysis methods are not used.

## Results

**Scheme 1:**
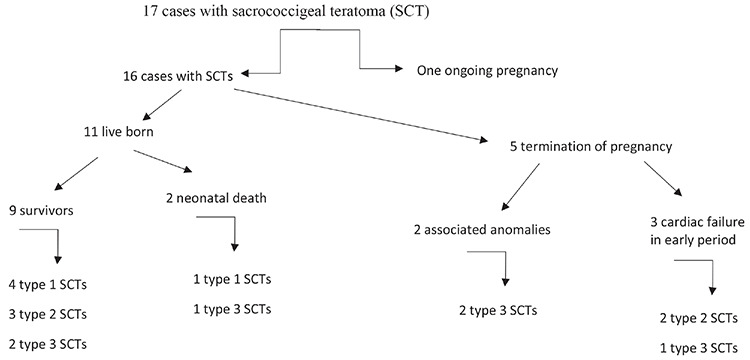
Seventeen cases with sacrococcygeal teratoma (SCT)

Out of a total of 18,500 fetal anatomy scans throughout the 10-year study period, SCT was detected in 17 pregnancies, revealing a 0.92/1000 incidence rate among high-risk cases in our tertiary setting. The gestational age at diagnosis ranged from 16 to 34 weeks (Table 1). Of the only two fetal karyotypes performed, both were normal. Five of the SCT pregnancies underwent termination of pregnancy. The indication for medical termination was early-onset cardiac failure (n=3) and additional fetal central nervous defects (n=2). Three fetuses had hydronephrosis due to the urinary obstruction (all with type 3 SCT). Two fetuses had fetal cardiomegaly and fetal cardiac failure due to the hyperdynamic flow, and these two fetuses died in the neonatal period. During cesarean section, a classic incision was required in one case, and lower segment transverse incision was used in the others. Almost half of the survivors had type 1 tumors. Nine out of 11 live born infants were alive at the time of writing. The cases are summarized in Scheme 1.

## Discussion

The incidence rate of SCTs in the present design was significantly higher than that reported previously, probably due to referrals to our tertiary setting. Population-based studies are required to delineate the true incidence of SCT in Turkey.

The prognosis of SCTs detected in the prenatal period is worse than those detected in the neonatal period^(14,15)^, explained by the fact that larger-sized tumors are more prone to be detected in fetal life. Tumors detected early in gestation may have a greater growth potential. The prognosis seems to deteriorate when the ratio of tumor volume to estimated fetal weight increases^(16,17)^. In the present study, cases diagnosed at earlier weeks of gestation were associated with adverse prenatal outcomes such as heart failure and termination of pregnancy. Hence, all fetuses with SCT can be monitored for the possible development of heart failure. However, there is no precise information about the most appropriate monitoring protocol because previous data are generally dependent on case series.

SCTs are not usually associated with chromosomal abnormalities. However, cases related to chromosomal abnormalities have also been described^(18,19,20,21)^, although this coexistence may be incidental. A previous report described tethered spinal cord associated with SCT^(22)^. In addition, there was no correlation between the ultrasonographic appearance (cystic or solid components) and the pathology of the tumor. The follow-up of our patients with immature teratoma is ongoing, and no complication has been observed to date.

Fetal magnetic resonance imaging (MRI) may be particularly useful in the differential diagnosis of SCT. Tumor type, solid component content, and tumor volume can also be detected successfully using MRI. However, the authors propose that fetal MRI may be unnecessary except for clinical studies. In the author’s opinion, tumor type, blood flow into the tumor, placenta size, and amount of amniotic fluid can be successfully demonstrated with ultrasound.

Tumor morphology is also important in prognosis. In some small case studies, solid tumors have been reported to have a worse prognosis than cystic tumors^(15,23,24)^. There is an increased risk of fetal cardiac insufficiency in solid tumors. These solid tumors may tend to be more vascular and cause more shunting of blood away from the placenta because they grow larger. Solid tumor volumes and derived indices are predictive of mortality and high-output cardiac failure^(12,25)^. We did not measure the tumor solid component volume in our cases, but we confirmed that the prognosis was worse in tumors with solid components.

Although there is no consensus on the mode of delivery, the authors recommend cesarean delivery except for early pregnancy termination or very early preterm deliveries. Tumor rupture during delivery can cause bleeding in varying proportions depending on the amount of vascularization of the tumor. It should be kept in mind that larger tumors may require classic uterine incisions. In the presence of a large tumor, an improper incision may cause detrimental tears in the uterus, leading to postpartum hemorrhage.

### Study Limitations

There are some limitations of the present study. First, the study was designed retrospectively. However due to the rarity of the tumor, information on this subject is usually based on retrospective case series. Recommendations such as approach and follow-up frequency in these cases are not based on randomized studies but as an expert opinion. Second, autopsy was declined in most of the cases. When an autopsy is not performed, a definitive pathologic diagnosis of a tumor considered as teratoma with ultrasound cannot be confirmed. Therefore, a false-positive rate cannot be given. Another significant limitation is the short follow-up period, especially in immature teratoma cases. Despite these, the most important advantage of our study is the high number of cases of a sporadic rare tumor.

## Conclusion

When a tumor is detected in the sacrococcygeal region, the differential diagnosis including SCT should include an appropriate evaluation. With serial examinations, it is necessary to determine the course of the disease and whether it will lead to heart failure.

## Figures and Tables

**Table 1:**
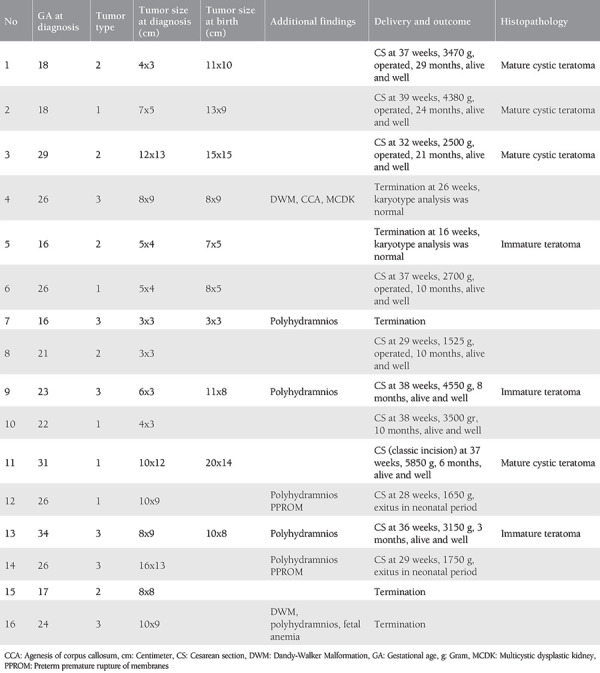
Associated anomalies, clinical features, and outcomes in 16 cases with sacrococcigeal teratoma
